# Determination of Mercury in Ayurvedic Dietary Supplements That Are Not Rasa Shastra Using the Hydra-C Direct Mercury Analyzer

**DOI:** 10.1155/2013/628397

**Published:** 2013-04-28

**Authors:** Amir A. Abdalla, Robert E. Smith

**Affiliations:** Total Diet and Pesticide Research Center, U.S. Food and Drug Administration, 11510 W 80th Street, Lenexa, KS 66214, USA

## Abstract

Mercury has been determined in Ayurvedic dietary supplements (Trifala, Trifala Guggulu, Turmeric, Mahasudarshan, Yograj, Shatawari, Hingwastika, Shatavari, and Shilajit) by inductively coupled plasma-mass spectrometry (ICP-MS) and direct mercury analysis using the Hydra-C direct mercury analyzer (Teledyne Leeman Labs Hudson, NH, USA). Similar results were obtained from the two methods, but the direct mercury analysis method was much faster and safer and required no microwave digestion (unlike ICP-MS). Levels of mercury ranged from 0.002 to 56 **μ**g/g in samples of dietary supplements. Standard reference materials Ephedra 3240 and tomato leaves that were from the National Institute of Standard and Technology (NIST) and dogfish liver (DOLT3) that was from the Canadian Research Council were analyzed using Hydra-C method. Average mercury recoveries were 102% (RSD% 0.0018), 100% (RSD% 0.0009), and 101% (RSD% 0.0729), respectively. Hydra-C method Limit Of Quantitation was 0.5 ng.

## 1. Introduction

 Ayurveda is a traditional form of medicine that originated on the Indian subcontinent during the Vedic period and is still widely used by many [1]. These “medicines” can be divided into two broad disciplines: herbal only and *rasa shastra*, in which metals, including mercury, are added [2]. In a survey of Ayurvedic medicines in the Boston area, 20% were found to contain lead, mercury, and/or arsenic when analyzed by X-ray fluorescence [3]. The range of mercury found was 28–104000 *μ*g/g [3]. Similar results were found in products obtained over the internet, in which 20% of all the products contained lead, mercury, and/or arsenic and 40.6% of the *rasa shastra* products contained mercury, ranging from 13 to 28 mg/g [4]. These are over four orders of magnitude greater than the FDA action level for mercury in fish, which is 0.001 mg/g or 1 *μ*g/g [5]. However, uncontrollable matrix effects make X-ray fluorescence a nonquantitative technique.

There are also metallic-herb preparations called bhasmas, which are used in the *rasa shastra* discipline [6]. Bhasma means “ash” and is a preparation made from precious metals and their salts [6]. One of the, Siddha Makaradhwaja, was found to contain 85.3 ± 7.0% mercury as HgS, with no traces of any other elements [6]. Another mercury-based bhasma, parad, contained 0.018 ± 0.002% (180 ± 2 *μ*g/g) [6]. Several other bhasmas that were not based on mercury still contained much more than 1 *μ*g/g, with one (Praval Pishti) containing 627 ± 20 *μ*g/g, when analyzed by flame atomic absorption spectrophotometry [6].

Other dietary supplements have been analyzed by ICP-MS for As, Pb, Hg, and Cd [7, 8], in which samples were prepared by microwave digestion [9]. This method is labor-intensive and has caused extreme safety concerns, including explosions [10]. There is another method based on oxidation, purge and trap, and cold vapor atomic fluorescence spectrometry that does not require digestion or any other sample preparation and it is the basis for US EPA method 7473 [11, 12]. It provides an inexpensive, simple, and convenient approach to mercury analysis without any sample pretreatment and without any hazardous chemical waste. Typical analysis times are approximately five min per sample. Solid samples are decomposed by combustion in the presence of O_2_. The gases produced are carried through a heated tube containing a catalyst, which removes halogens, nitrogen oxides, and sulfur oxides. The remaining combustion products, which include Hg^0^, are swept through a gold amalgamation tube that captures the Hg^0^ and then releases it as a gaseous bolus into the oxygen carrier gas, which sends it to a direct mercury analysis. The transient signal is measured in series by high and low sensitivity cells. The two peaks are integrated and compared to the best calibration data of the two cells. This provides a wider dynamic range (3–600 ng) than that of a single optical cell analysis. US EPA Method 7473 method was used in the present study to determine Hg in different samples of Indian dietary supplements that were not *rasa shastra*. They included Trifala, Trifala Guggulu, Turmeric, Mahasudarshan, Yograj, Shatawari, Hingwastika, Shatavari, and Shilajit. Trifala (or triphala) has been reported to be a 1 : 2 : 3 mixture of the three fruits called Harr, Bahera, and Aonla [13]. Trifala guggulu has been reported to be a guggulu-based formulation, in which the guggulu is an oleoresin from the *Commiphora mukul* tree [14]. Turmeric is the rhizome of *Curcuma longa* and contains the compound curcumin, which has many health benefits [15]. Mahasudarshan is a preparation of tablets containing 45 ingredients, one sample of which was found to have only 0.07 *μ*g/g Hg [16]. Yograj has been reported to have 28 ingredients and is often sold as a mixture with guggulu [17]. Shatawari (or Shatavari) is made from the root tubers of *Asparagus racemosus* Linn [18]. Hingwastika has been reported to only contain herbs, one sample of which was found to have 37 *μ*g/g Hg [19]. Shilajit has been reported to be an exudate from rocks in the Himalaya mountains and it contains mostly paleohumus and organic compounds from fossilized plants [20].

## 2. Materials and Methods

### 2.1. Reagents and Samples

Samples of Ayurvedic dietary supplements were purchased over the internet. Standard reference materials Ephedra 3240 and tomato leaves were from NIST and dogfish liver (DOLT3) was from the Canadian Research Council. Approximately half of each bottle of dietary supplements sample was placed in a blender and then blended to homogenous composite prior to the analysis. For products provided in tablet form approximately, half of each bottle was manually grounded into homogenous composite. Trace metals grade 30% H_2_O_2_, concentrated HCl, HNO_3_ and concentrated H_2_SO_4_ were purchased from GFS (Powell, OH) and used for the digestion. A 1000 *µ*g/mL Hg standard in 2% HNO_3_, from ICP International (Santa Rosa, CA, USA), was used to spike the samples and measure spike recovery.

### 2.2. Sample Preparation

Microwave digestion of about 0.7 g of sample was done using Milestone Ultrawave microwave digestion system (Shelton, CT, USA) and quartz tubes. The microwave system contains a large pressurize reaction chamber in which all samples were digested simultaneously. The reaction chamber was prepressurized using N_2_ gas prior to start the run. Power was 1500 Watts, with a starting temperature of 120°C and pressure at 40 bar and then the temperature increased linearly to 230°C for 8 min and then held at 230°C for 8 min. The reaction chamber was cooled to 25°C. After cooling to 25°C, a final 0.5 mL of 30% H_2_O_2_, and 0.5 mL of HCl were added to each sample, followed by diluting to 100.0 mL with deionized water. Acids concentrations in sample digests were approximately 2% HNO_3_ and 0.5% HCl (HCl was used to stabilize Hg). ICP-MS analysis was done on an Agilent 7700x ICP-MS (Santa Clara, CA, USA). Spiking standards were prepared by adding Hg from a stock standard solution of 1000 *µ*g/mL Hg in 2% HNO_3_, from ICP International (Santa Rosa, CA, USA) to a flask containing a solution of 5% nitric acid and 1.0% HCl to a final concentration of 500 *μ*g/L. The standard was added to the solid samples in the sample boat. Iridium was used as the internal standard. The percent recoveries of samples spiked with Hg are listed in Table 1. Isotopes of Hg and internal standard for ICP-MS analysis are listed in Table 2. ICP-MS instrument was calibrated using NIST traceable standards. The calibration has a correlation coefficient (*r* ^2^) value of >0.995.

### 2.3. Instrumental Analysis

For the determination of Hg, a Hydra-C mercury analyzer, model IIc was used (Teledyne Leeman Labs Hudson, NH, USA) without any sample digestion. Approximately 100 mg of each dietary supplement sample (60–85 mg National Institute of Standard and Technology (NIST) Standard Reference material (SRM) 3240 (*Ephedra sinica* Stapf Aerial Parts), tomato leaves, and dogfish liver (Gaithersburg, MD, USA) was weighed directly into a nickel boat, and then the nickel boat was introduced into a decomposition furnace. Percent recoveries were measured in duplicate. The thermal decomposition technique described in EPA method 7473 and ASTM method 6722-01 was used for the analysis. The Standard Operating Conditions for Direct Mercury Analysis are listed in Table 3.

Prior to sample analysis Hydra-C instrument was calibrated using NIST traceable standards. Calibration was completed using aqueous standards prepared in 1.0% HNO_3_. Instrument linearity was established across two separate ranges; a low range (0 ng to 30 ng) and high range (40 ng to 600 ng). The high calibration range has a correlation coefficient (*r* ^2^) value of 0.9992 and the low calibration range has an *r* ^2^ value of 0.9996. Six dietary supplements samples were fortified with approximately 0.10 ng/g Hg to check for matrix effects. The percent recoveries of samples spiked with Hg are listed in Table 1.

## 3. Results and Discussion

The two methods gave very similar results for the ten samples analyzed, as shown in Table 4. This is much like the agreement between results that was seen in red yeast rice [21]. Levels of mercury ranged from 0.002 to 56 *μ*g/g. Even though the two methods found different amounts in the sample containing the most Hg, both methods can easily distinguish between samples that are below or above the 1 *μ*g/g action level. It should be noted that the sample containing the most Hg had to be diluted by adding some of the stock standard solution to the solid sample, which can cause errors due to sample inhomogeneity. As reported by others [19], it is important to dilute the sample before analyzing it, to prevent contaminating the instrument with excess Hg.

As a test of accuracy, Hydra C method found the amounts of Hg in standard reference materials (Table 5), and spiked samples (Table 1) that were close to the expected values. ICP-MS method found the proper amounts of Hg in spiked samples (Table 1). Therefore, it can be concluded that the much faster and safer method using the Hydra C can provide similar accuracy as the ICP-MS method that requires microwave digestion of the samples. Moreover, four of the ten samples had much more than 1 *μ*g/g of Hg. A comparison and practical considerations for using Hydra C versus ICP-MS technique for mercury analysis are listed in Table 6.

## Figures and Tables

**Table 1:** Percent recoveries of Hg spikes by ICP-MS and Hydra C.

Item	Name	ICP-MS percent recovery	Hydra C percent recovery
Tu-S1	Turmeric	106%	110%
Shv-S1	Shatawari	112%	112%
Shil-S1	Shilajita	93%	106%

**Table 2:** Isotopes of Hg for ICP-MS, used with an iridium internal standard.

Element	Analyte mass	Internal standard mass
Hg	201	193
Hg	202	193

**Table 3:** Standard operating conditions for Hydra-C direct mercury analyzer.

Parameter	Time (s)	Temp (°C)
Dry	50	250
Decomposition	200	600
Catalyst	60	600
Amalgam	30	600
Integration	60	N.A.
Oxygen flow	300 ml/min	N.A.

**Table 4:** Comparison between Hydra C and ICP-MS results, measured in duplicate.

Item	Hydra-C (*μ*g/g)	ICP-MS (*μ*g/g)
Trifala Guggulu	5.109	4.345
Trifala	0.188	0.182
Turmeric	0.006	0.004
Mahasudarshan	9.519	10.644
Yograj	8.233	9.766
Shatawari	0.013	0.011
Hingwastika	38.959	56.503
Shatavari	0.007	0.006
Triphala	0.002	0.005
Shilajita	0.760	0.686

Trifala and Triphala are two different samples of what may be the same dietary supplement.

**Table 5:** Analysis of standard reference materials (SRMs) by the Hydra C for Hg.

QC Standards	Certified Hg content (*μ*g/g)	% recovery
SRM 3240 ephedra	0.0167 ± 0.0005	101 ± 10
SRM tomato leaves	0.0340 ± 0.004	100 ± 2
SRM DOLT 3 dogfish liver	3.37 ± 0.14	101 ± 3

**Table 6:** Comparison and practical considerations for using Hydra C versus ICP-MS.

Hydra C	ICP-MS
No Sample digestion (lower cost per analysis)	Need sample digestion
No Hazardous chemical or waste	Hazardous chemical or waste
All of the Hg in each sample is collected on the amalgam prior to analysis	Hg can be lost from aqueous samples during digestion
About 5 minutes/sample	About 30 min/sample
All the Hg gets to the detector, so sensitivity is good.	ICP-MS system must provide very high sensitivity to compensate for Hg low degree of ionization in the plasma
Decrease the chance of contamination	Chance of contamination during the digestion process
No carry over effect or loss of sample (no tubing required)	Significant carry over effect and loss of sample via tubing.
